# Kv4.2 Mediates Histamine Modulation of Preoptic Neuron Activity and Body Temperature

**DOI:** 10.1371/journal.pone.0029134

**Published:** 2011-12-29

**Authors:** Jasmine Sethi, Manuel Sanchez-Alavez, Iustin V. Tabarean

**Affiliations:** The Department of Molecular and Integrative Neurosciences, The Scripps Research Institute, La Jolla, California, United States of America; University of Bristol, United Kingdom

## Abstract

Histamine regulates arousal, circadian rhythms, and thermoregulation. Activation of H3 histamine receptors expressed by preoptic GABAergic neurons results in a decrease of their firing rate and hyperthermia. Here we report that an increase in the A-type K^+^ current in preoptic GABAergic neurons in response to activation of H3 histamine receptors results in decreased firing rate and hyperthermia in mice. The Kv4.2 subunit is required for these actions in spite of the fact that Kv4.2−/− preoptic GABAergic neurons display A-type currents and firing characteristics similar to those of wild-type neurons. This electrical remodeling is achieved by robust upregulation of the expression of the Kv4.1 subunit and of a delayed rectifier current. Dynamic clamp experiments indicate that enhancement of the A-type current by a similar amount to that induced by histamine is sufficient to mimic its robust effect on firing rates. These data indicate a central role played by the Kv4.2 subunit in histamine regulation of body temperature and its interaction with pERK1/2 downstream of the H3 receptor. We also reveal that this pathway provides a mechanism for selective modulation of body temperature at the beginning of the active phase of the circadian cycle.

## Introduction

The neurons of the tuberomammillary nucleus represent the main source of histamine in the brain. They project their axons throughout the brain and control arousal, attention, feeding, circadian rhythms and thermoregulation (reviewed in [1]). The preoptic area/anterior hypothalamus (PO/AH), region which contains thermoregulatory neurons as well as dense histaminergic projections appears to be the main locus in which histamine affects body temperature [2]. Recent studies have revealed that preoptic GABAergic neurons play an important role in the control of thermoregulatory networks that comprise also neurons of the rostral raphe pallidus (rRPa) and dorsomedial hypothalamus (DMH) (reviewed in [3]). We have previously reported that histamine influences body temperature by activating H1 and H3 receptors expressed by glutamatergic and GABAergic preoptic neurons, respectively [4].The mechanisms involved in these alterations of firing activity are not known. We focus in this study on histamine signaling in GABAergic neurons of the median preoptic nucleus (MnPO) because of their critical role in several thermoregulatory responses [5]–[8].

H3 subtype receptors (H3R) are present at presynaptic terminals and have inhibitory actions on the release of various neurotransmitters in the brain (reviewed in [9]). The signaling pathways activated downstream of H3 receptors involve either PKA, the extracellular regulated MAP kinase (ERK) or Ca^2+^ release from intracellular stores [10]–[12]. Several studies have revealed also postsynaptic inhibitory effects of H3R activation [4], [13], [14] however the signaling mechanisms involved are not known. Here we have tested the hypothesis that H3R modulation of A-type K^+^ currents (I_A_) mediates histamine effects on the firing activity of preoptic GABAergic neurons and on core body temperature (CBT).

## Materials and Methods

### Ethics statement

All animal work was conducted in accordance with the Institutional Animal Care and Use Committee of the Scripps Research Institute (approval ID #08-0129). The standards are set forth by American Association for the Accreditation of Laboratory Animal Care (AAALAC) standards and the regulations set forth in the Animal Welfare Act.

### Slice preparation

Coronal tissue slices containing the median preoptic nucleus (MnPO) were prepared from C57/Bl6 or Kv4.2−/− male mice (28–42 days old) housed in standard conditions. The Kv4.2−/− mice were a kind gift from Dr Jeanne Nerbonne (Washington University, St. Louis). These transgenics have been generated on the C57/Bl6 background. Acute slices containing the median preoptic nucleus (MnPO) were prepared as described previously [4]. The slice used in our recordings comprised the sections located from 0.5 mm to 0.25 mm from Bregma in the mouse brain atlas [15] and corresponded to the location of the canulla in our *in vivo* experiments (see below). The *in vivo* injections (a volume of 0.2 µL) were directed to the MnPO (see below), however some spread to nearby MPA neurons is expected. Patch-clamp recordings were made from neurons in the median preoptic nucleus (MnPO) and the adjacent medial preoptic area (MPA). Results from MnPO and MPA neurons were similar and therefore were pooled. The recorded cells were referred to as “preoptic neurons”. The slices were prepared at 9–11 am local time during the “subjective light period” and recordings were carried out up to the end of this period i.e. 8 pm local time.

### Identification of GABAergic preoptic neurons from w-t and Kv4.2−/− mice

**Figure 1:**
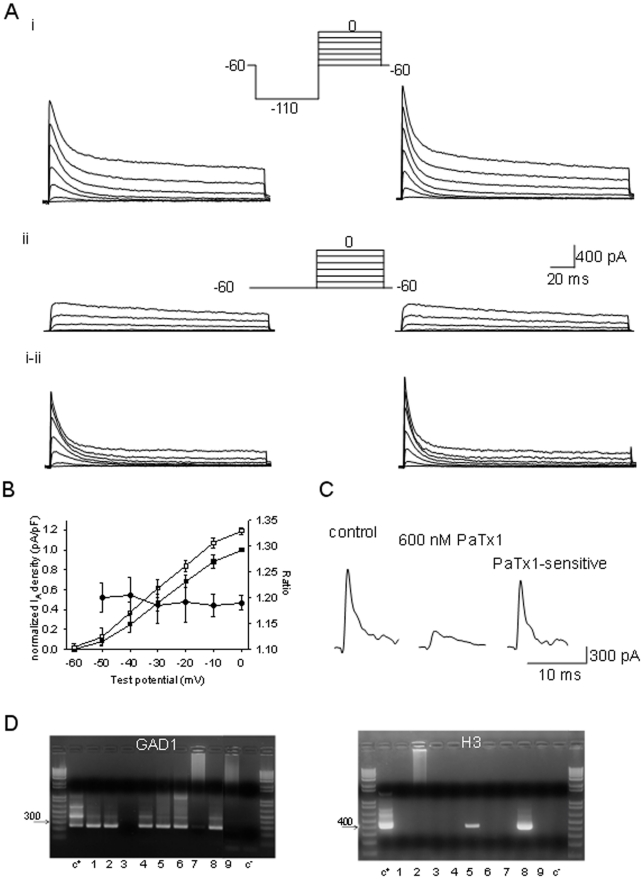
H3R activation increases I_A_ in preoptic GABAergic neurons. (i) Voltage-gated K^+^ currents recorded in the presence of TTX in control (left) and after 4 min incubation with R-α-methylhistamine (1 µM) (right). The currents were elicited by the voltage step protocol depicted in the inset. (ii) I_DR_ elicited by the voltage step protocol depicted in the inset before (left) and during R-α-methylhistamine (1 µM) incubation (right). (i–ii) Separation of I_A_ by subtraction of I_DR_ from the total voltage-gated K^+^ current. Note the increase in I_A_ amplitude during R-α-methylhistamine (1 µM) incubation (right). B. Voltage dependence of the normalized peak I_A_ amplitude in control (•), during H3 agonist incubation (○), and of their ratios (▪). Control currents were normalized relative to the value at 0 mV. The peak currents during H3 agonist incubation were normalized to the respective control. Pooled data from 6 neurons presented as means ± S.D. C. K^+^ current elicited by a depolarizing step to −20 mV in control (left), during PaTx1 (600 nM) incubation (middle) and the PaTX1 sensitive component obtained by subtraction (right). D. Agarose gels illustrating the expression of GAD1 (left) and H3R (right) in a batch of nine (1–9) recorded preoptic neurons. The expected sizes of the PCR products are (in base pairs) 244 and 403, respectively. Negative (−) control was amplified from a harvested cell without reverse-transcription, and positive control (+) was amplified using 1 ng of hypothalamic mRNA.

GABAergic neurons represent a large proportion of MnPO neurons and are characterized by pacemaking activity and higher firing rates (above 6–7 Hz) than those of non-GABAergic neurons [4]. We have used this criterion for preliminary identification of GABAergic neurons, however only neurons in which the expression of the GABAergic marker GAD1 was subsequently confirmed were included in this study. For that the cytoplasm of the recorded neurons was aspirated at the end of the experiment and stored at −80 C. The cytoplasm samples were analyzed by RT/PCR (as described below) in batches of 6 to 10 within 5 days after being harvested. Fig. 1D illustrates such a test in which 6 out of 9 recorded neurons were confirmed GAD1-positive.

### Dissociated preoptic neurons from slices

The MnPO was punched out of a brain slice and incubated in Hibernate A and papain (1 mg/ml) for 10 min. After washing out the enzyme with Hibernate –A the cells were dissociated by gentle trituration with a fire-polished Pasteur pipette. The cell suspension was pelleted (1000 g for 2 min) and resuspended in Neurobasal medium and then plated on coverslips (Biocoat, BD Biosciences, Belgium). Cells were allowed to attach to the coverslips for 2–3 hours before recording. For immunocytochemistry cells were kept in culture for 7–10 days to allow them to firmly attach to the coverslips.

### Whole-cell patch clamp recording

The artificial cerebrospinal fluid (aCSF) contained: 130 NaCI, 3.5 KCI, 1.25 NaH_2_PO_4_, 24 NaHCO_3_, 2 CaCI_2_, 1 MgSO_4_, 10 glucose osmolarity of 300–305 mOsm, equilibrated with 95% O_2_ and 5% CO_2_ (pH 7.4). Other salts and agents were added to this medium. In some experiments the aCSF was supplemented with CNQX (20 µM) and picrotoxin (50 µM) to block fast synaptic events. A K^+^ pipette solution containing 130 K-gluconate, 5 KCI, 10 HEPES, 2 MgCI_2_, 0.5 EGTA, 2 ATP, 1 GTP (pH7.3) was used in most experiments. Glass micropipettes were pulled with a horizontal puller (Sutter Instruments) using Sutter borosilicate glass capillaries with filament. The electrode resistance after back-filling was 2–4 MΩ. All voltages were corrected for the liquid junction potential (−13 mV). R-α-methylhistamine was from Tocris (Ellisville, MO), phrixotoxin 1 (PaTx1) was from Alomone Labs (Jerusalem, Israel), while the other substances were purchased from Sigma. The recording chamber was constantly perfused with aCSF (2–3 mL/min). The treatments were applied locally using a perfusion pencil system (tip diameter 100 µm, Automate Scientific) driven by gravity.

### Temperature control

The temperature of the external solution was controlled with a TC-344B temperature controller and an inline heater (Warner Instruments, Hamden CT) and was maintained at 36–37°C.

### Data acquisition and analysis

Data was acquired with a MultiClamp 700B amplifier digitized using a Digidata 1320A interface and the Pclamp9.2 software package (all from Molecular Devices, Sunnyvale, CA). The sampling rate for the continuous recordings of spontaneous activity was 50 kHz. After establishing whole-cell configuration the spontaneous activity of the neuron was recorded for 2–4 min to determine its control behavior at (36–37°C). The spontaneous firing activity of the neurons was recorded in whole-cell mode (current clamp, I = 0). The cell capacitance was determined and compensated using the Multiclamp Commander software. The spontaneous firing rates (averaged over 10 s) as well as the K^+^ currents were very stable over ∼30 min recordings in control extracellular solution displaying spontaneous changes of less than ±3%. Therefore all changes larger than ±3% in response to a pharmacological treatment were considered “responses”. Action potential parameters were determined using Mini Analysis software in which the threshold was defined as the point in which dV/dt exceeds 4 Vs^−1^. The amplitude of the afterhyperpolarization (AHP) was measured as the difference between spike threshold and the most negative voltage reached during AHP. The slope of decay of the AHP was measured as the voltage change over 5 ms following the most negative voltage reached.

### Dynamic clamp

The dynamic clamp functionality of the QUB software (http://www.qub.buffallo.edu/milescu) developed by Dr Lorin Milescu [16] was run on Dell T7400 computer equipped with a Intel Xeon quadcore processor. A National Instruments PCI 6251 card was used for data acquisition and for generating the dynamic clamp current. The sampling rate was 50 kHz. The real time performance of the dynamic clamp system was adequate, with only 1 in 10^5^ cycles lasting more than 20.1 µs. I_A_ was modeled using the Hodgkin and Huxley formalism I_A_ = g_A_m^4^h(V−V_rev_). Where m and h are the occupancy probabilities for the activation and inactivation particles, respectively. dm/dt = α_m_* (1−m)−β_m_*m and dh/dt = α_h_*(1−m)−β_h_*h. The gating transitions are described by the rate constants that have Eyring voltage-dependencies: α = α_0_*exp α_1_*(V−α_0.5_), β = β_0_*exp β_1_*(V−β_0.5_) [16]. Starting from values determined for other recordings (http://www.qub.buffallo.edu/milescu) we have empirically determined values that fit our recorded I_A_ currents. The respective parameters were: α^m^_0_ = 9, α^m^_1_ = 0.059, α^m^_0.5_ = 0, β^m^_0_ = 0.01, β^m^_1_ = −0.1, β^m^_0.5_ = −10, α^h^_0_ = 0.001, α^h^_1_ = −0.1, α^h^_0.5_ = −25, β^h^_0_ = 0.3, β^h^_0.5_ = 0. β^h^_1_ was changed among neurons to account for the differences in inactivation rate and ranged from 0.10 to 0.15. We have tested our models online using a model cell (Patch-1U, Molecular Devices) attached to the second stage of the Multiclamp 700B: step depolarizations from a holding potential of −110 mV were applied and the currents elicited by the dynamic clamp where compared with those recorded in the modeled neuron. The two traces were compared using a minimum sum of squared errors.

### Immunocytochemistry and confocal imaging

Coverslips were washed by immersion in HBS, fixed in ice-cold 4% paraformaldehyde for 30 min, and then incubated for 10 min at room temperature in PBS containing 0.25% Triton X-100. Nonspecific sites were blocked by incubation in PBS containing 10% normal goat/horse serum. Antibodies against Kv4.2 and Kv4.3 were from Neuromab (UC Davis), the Kv4.1 antibody was from Alomone Labs (Jerusalem, Israel), the H3 and the GAD67 antibodies were from Invitrogen (Temecula, CA). The specificity of the antibodies was checked by the supplier and by previous studies by others. We have confirmed the specificity of the Kv4.2 antibody by staining Kv4.2−/− neurons. Specific binding was detected by using secondary antibodies conjugated to AlexaFluor dyes (594, red; 488, green; Molecular Probes). Images were collected on a Delta Vision Optical Sectioning Microscope (Olympus, Melville, NY). Multiple consecutive optical sections at a 0.24 µm interval were acquired for each of the fluorescent probes by using a 60× oil objective. The settings of the confocal were kept constant so that the images could be analyzed by densitometry and the different conditions could be compared. 15 different fields in each coverslip were captured, and all neurons in the pictures were analyzed for their green and red fluorescence content. Backgrounds were defined as the fluorescence intensity in non-neuronal fields and subtracted from the values obtained for each neuron. Using Image J software (NIH) we have quantified the intensity of the fluorescent signal as integrated density.

### Cell harvesting and reverse transcription

Preoptic neurons in slices or dissociated were patch-clamped and then harvested into the patch pipette by applying negative pressure. The content of the pipette was expelled in a PCR tube. dNTPs (0.5 mM), 50 ng random primers (Invitrogen) and H_2_O were added to each cell to a volume of 16 µl. The samples were incubated at 65°C for 5 min and then put on ice for 3 min. First strand buffer (Invitrogen), DTT (5 mM, Invitrogen), RNaseOUT (40 U, Invitrogen) and SuperScriptIII (200 U, Invitrogen) were added to each sample to a volume of 20 µl followed by incubation at room temperature for 5 min, at 50°C for 50 min and then at 75°C for 15 min. After reverse transcription samples were immediately put on ice. 1 µl of RNAse H was added to samples and kept at 37°C for 20 min.

### Nested PCR

Gene specific primers for GAD1 and histamine receptor H3 were as described elsewhere [4]. cDNAs were amplified in a volume of 25 µl using a high-fidelity Platinum Taq polymerase kit (Invitrogen) and 0.4 mM dNTPs (Invitrogen). For each gene 1/10 of the cDNA from each cell was subjected to a first round of PCR using the outer primer pair and a thermal cycling program with an initial denaturation at 94°C for 2 min, 35 cycles of denaturation at 94°C for 15 s, annealing at 55°C for 30 s and extension at 68°C for 45 s followed by a final extension at 68°C for 10 min. 1.5 µl of the PCR product was subjected to a second round of PCR using the inner primer pair and 40 cycles of the same thermal cycling program as above with the extension time reduced to 30 s. PCR products were visualized by ethidium bromide stained 2% agarose gel electrophoresis.

### RT/QPCR

Preoptic tissue obtained from slices was stored at −80 degree Celsius until the time of RNA extraction. Total RNA was extracted using Qiagen RNAeasy kit manufacturer's instructions. RNA extraction was conducted in dry ice. The concentration of RNA was determined using a nanodrop measuring the absorbance at 260 nm with reference to 280 nm. RNA was then stored at −80 degree Celsius. 4 µg RNA was then reverse-transcribed into first-complimentary DNA (cDNA) using Superscript III reverse transcriptase kit (Invitrogen, CA) according to manufacturer's instructions. The reaction was inactivated by incubating it at 75°C for 10 minutes. In order to remove RNA complimentary 1 µl RNAse H was added to the reaction and was incubated at 37°C for 20 minutes. The concentration of prepared cDNA was measured using nanodrop measuring absorbance at 230 nm with reference to 280 nm. Quantitative PCR reactions were performed in duplicate using Roche light-cycler and the following parameters were used for 45 cycles (95°C for 10 minutes, 95°C for 5 seconds, 60°C for 5 seconds, 72°C for 8 seconds for 45 cycles; for melting: 95°C for 5 seconds, 65°C for 5 seconds; for cooling 40°C for 30 seconds). Each 20 µl of PCR reaction contained: 4 µl Sybr green (Roche, enzyme mixed according to manufacturer's instructions), 9 µl PCR graded water, 2 µl 1 µM gene of interest primer and 5 µl diluted cDNA (1/30 dilution). Each reaction included a standard curve. To determine a standard curve, 1 µl of the non- diluted cDNA for all samples were mixed and a dilution of 1∶100, 1∶50 and 1∶ 25 was made. The standard curve is used for determining the efficiency of the primer and calculating the concentration of the mRNA amplified in the QPCR reaction. The concentration of each gene was normalized using the housekeeping gene GAPDH. Primers used were: Kv4.1 (NM_008423) sense 5′-GCTGCTCTCGAAGGGTCAAT-3′, antisense 5′-CACGGCTGACAGAGGCAGTA-3′, Kv4.3 (NM_001039347) sense 5′- CCTGCTGCTCCCGTCGTA-3′, antisense 5′- GGGTGGCAGGCAGGTTAGA-3′.

### Radiotelemetrv studies of core body temperature and motor activity

Mice were implanted with the radio transmitter (TA10TA-F20™ and RPC-1™, Data Sciences, Inc.) as previously described [4]. The cages were positioned onto the receiver plates. Radio signals from the animals' core body temperature (CBT) and motor activity (MA) were continuously monitored by a fully automated data acquisition system (Dataquest™ A.R.T.™, Data Sciences, Inc). C57/Bl6 and Kv4.2−/− adult males (12–14 weeks old) were used for the *in vivo* experiments.

### Stereotaxic injections

Cannula implantation and injections were carried out as previously described [4]. Coordinates for cannula (27 ga. 16 mm length) implants in the median preoptic nucleus (MnPO): from Bregma: 0.38 mm and ventral 4.6 mm [15]. The ambient temperature was maintained at ∼25±0.5°C in a 12:12-h lightdark cycle controlled room (lights on 8:00 am). H3R ligands and the MEK-1 inhibitor PD98059 were injected into the MnPO. All substances injected were dissolved in sterile aCSF. Mice were handled for at least three days before the injection during 5 minutes every day for habituation. On the day of injections, mice were held and the injector (cannulae 33 Ga, 17 mm length) was placed inside the cannula. The injector was connected to a microsyringe (1.0 µL). The injected volume was 0.2 µL (rate 0.1 µL/min). After this procedure the animal was returned to the home cage. Injections were always performed at 12 am local time, during the “subjective light period”.

### Histology

The cannula placement was always checked at the end of the experiments by blue pontamine dye injection. The animals were then killed and the brains were removed and cryosectioned. The cannula placement in the MnPO was confirmed using an inverted microscope.

### Statistics

The values reported are presented as mean ± standard deviation (S.D.). Statistical significance of the results pooled from two groups was assessed with *t*-tests using Prism4 (GraphPad Software). Non-parametric one way analysis of variance (ANOVA, Kruskal-Wallis) with Dunn's post hoc test (*P*<0.05) was used for comparison of multiple groups. Comparisons of cumulative distributions were done with Kolmogorov-Smirnov test (*P<0.05*). All data collected as time series were compared across genotypes and time points by two way ANOVA with repeated measures (*P*<0.05), followed by unpaired *t*-tests (*P*<0.05) for comparisons at each time point (Prism4, GraphPad Software).

## Results

### Activation of H3R receptors increases the A-type K^+^ current in preoptic GABAergic neurons

We have previously reported that activation of H3R histamine receptors results in a potent decrease in the firing rate of preoptic GABAergic neurons, effect that was not dependent on synaptic activity or changes of resting membrane potential [4]. We hypothesized that the agonist modulates the “pacemaking” mechanism. Therefore we tested the effect of the H3 agonist R-α-methylhistamine (1 µM) on voltage-gated conductances that are involved in rhythmic firing activity and have been identified in preoptic neurons: Na^+^ current, A-type K^+^ current (I_A_), delayed rectifier K^+^ current (I_DR_), L-type and T-type Ca^2+^ currents, and I_h_ current [17], [18]. GABAergic neurons were identified as described in Materials and Methods. I_A_ increased during incubation with the H3 agonist (Fig. 1A) in a population of GABAergic neurons while the other currents were not affected. The I_A_ was isolated by a voltage step protocol: the neuron was depolarized from a holding potential of −60 mV to test potentials between (−60 and +10) with or without a conditioning step to −110 mV. Without the conditioning step (that allows I_A_ to recover from inactivation) the elicited currents had no distinguishable transient component. By subtracting them from the currents obtained with the conditioning pulse we could separate I_A_, however a small I_DR_ was still present (Fig. 1A). To fully separate I_A_ we have added TEA (5 mM) and 4-AP (0.25 mM) (see below) to the extracellular solution. After measuring I_A_ twice in control conditions to verify its stability, we bath applied R-α-methylhistamine (1 µM) and within 3 minutes we noticed an increase in the current's amplitude (Fig. 1A). In contrast I_DR_ was not modified by the agonist. Similar results were obtained in 5 other GABAergic neurons out of 18 tested, reflecting the percentage of H3R expressing cells [4]. The increase in I_A_ amplitude averaged 18±4% (n = 6 out of 18, P<0.05, paired t-test relative to control amplitudes) at −20 mV test potential. The enhancement of I_A_ peak currents in the presence of the agonist was statistically significant (t-test, P<0.05) and of similar size at all voltages tested (Fig. 1B). In the remaining 12 neurons the agonist had no effect on either I_A_ or I_DR_.

To allow a faster exchange of extracellular solutions and to avoid space clamp limitations as well as possible presynaptic effects activated by pharmacological treatments we continued this study on acutely dissociated preoptic neurons from slices (see Methods). As observed in slices, R-α-methylhistamine (1 µM) induced an enhancement of I_A_ in a subpopulation of GABAergic neurons that averaged 18±5% (n = 10, out of 29 neurons studied) at −20 mV test potential. The percentage increase values were similar to those observed in neurons recorded in slices (p>0.5, t-test). The agonist was without effect on either I_A_ or I_DR_ in the remaining GABAergic neurons. To verify that the effect was due to activation of H3Rs we have tested the agonist after incubation with the H3R antagonist thioperamide (1 µM). In the presence of the antagonist, R-α-methylhistamine (1 µM) was without effect in all GABAergic neurons tested (n = 26). Finally, we have probed the expression of the H3R transcripts using single cell RT/PCR in cells in which R-α-methylhistamine (1 µM) effects on I_A_ were tested. In 4 out of 4 neurons in which the agonist increased I_A_ we have detected H3R transcripts. Conversely, H3R transcripts were not detected in any of the 21 neurons where the agonist had no effect. Fig. 1D illustrates such an analysis of a batch of 9 recorded neurons: 6 were GAD1-positive two neurons in which I_A_ was enhanced by the H3 agonist were found to be also H3R-positive, while the remaining neurons were H3R-negative.

Several subunits belonging to the Kv1, 3 and 4 families can form channels that conduct I_A_ currents. To identify the Kv subunits that contribute to I_A_ in preoptic GABAergic neurons we have first carried out pharmacological experiments. I_A_ was very sensitive to the Kv4-selective phrixotoxin-1 (PaTx-1)(600 nM). The amplitude of the current (at −20 mV test potential) reversibly decreased by 76±11% (n = 5, P<0.01 paired t-test relative to control amplitudes) in the presence of the toxin (Fig. 1 C). The toxin displayed similar potency at all voltages tested (−30 to 0 mV).

### Effects of H3R agonist on the spontaneous activity of w-t and Kv4.2−/− GABAergic preoptic neurons

**Figure 2:**
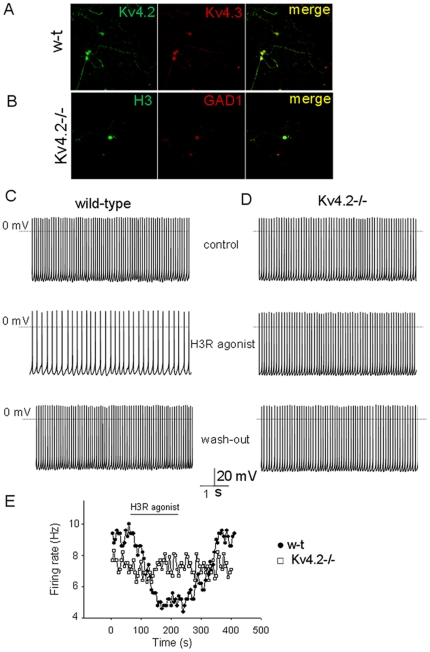
Activation of H3 subtype histamine receptors reduces the firing rate of preoptic w-t GABAergic neurons but has no effect on the firing rate of Kv4.2−/− neurons. A. Single channel confocal images showing immunoreactivity to Kv4.2 (*Left*, green) and Kv4.3 (*Middle*, red) and their superimposition (*Right*, merge) obtained from dissociated w-t preoptic neurons. B. Immunoreactivity to H3R (*Left*, green) and GAD1 (*Middle*, red) and their superimposition (*Right*, merge) obtained from dissociated Kv4.2−/− preoptic neurons. Note the immunoreactivity to both H3R and GAD1 of a Kv4.2−/− neuron. C, D. Expanded fragments of a recording showing “pacemaker” firing of the neuron in control (upper trace), during R-α-methylhistamine (1 µM) application (middle), and during washout (lower) from a w-t (C) and a Kv4.2−/− (D) dissociated neuron. E. Average firing rate (for every 10 s) recorded before, during, and after application of R-α-methylhistamine (1 µM). The filled circles (•) and squares (□) correspond to the experiment presented in B and C, respectively.

**Figure 3:**
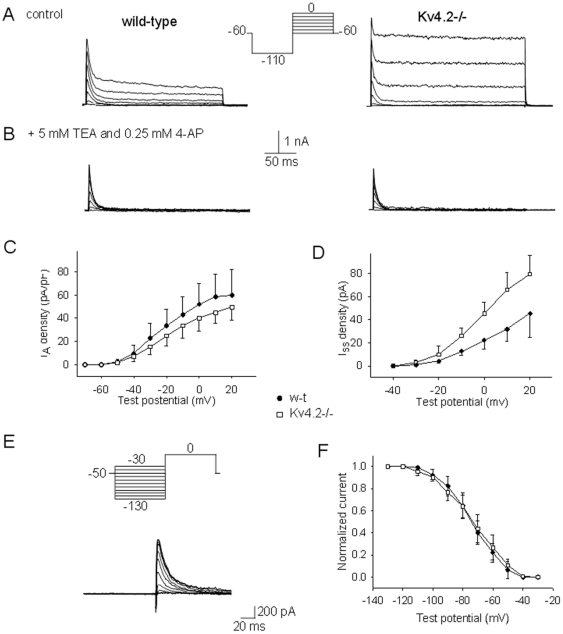
Voltage-gated K^+^ currents recorded in w-t and Kv4.2−/− preoptic GABAergic neurons. A. Voltage-gated K^+^ currents recorded in the presence of TTX (1 µM) in w-t (left) and Kv4.2−/− (right) neurons. The currents were elicited by the voltage step protocol depicted in the inset. Note the large I_DR_ present in the Kv4.2−/− neuron. B. Separation of I_A_ in the presence of TEA and 4-AP. The currents were elicited by the same voltage step protocol as in A. Recordings were from the same neurons as in A (upper traces). Lower traces represent the same traces on an expanded timescale. C. I–V plot for peak I_A_ amplitude in w-t (•) and Kv4.2−/− neurons (□) recorded in the presence of TEA and 4-AP. D. I–V plot for the steady state component of the current at the end of the depolarizing steps (Iss) in w-t (•) and Kv4.2−/− neurons (□) recorded in standard extracellular solution. C, D. Data points represent averages ±S.D. from n = 14 w-t neurons and n = 15 Kv4.2−/− neurons. The data points at −70, +10 and +20 mV are from a subset of n = 6 w-t neurons and n = 6 Kv4.2−/− neurons. E. I_A_ currents recorded in the presence of TEA and 4-AP in response to the voltage step protocol depicted in the inset. A depolarizing step to 0 mV was preceded by a 200 ms conditioning step to voltages ranging from −130 to −30 mV. F. Voltage-dependence of inactivation of I_A_ recorded in w-t (•, n = 8) and Kv4.2−/− neurons (□, n = 8). Data presented as averages ± S.D.

Kv4.2 and Kv4.3 subunits are highly expressed in many regions of the brain, including in the hypothalamus, in contrast to Kv4.1 subunits that are present only at low levels. Immunocytochemistry experiments on preoptic neurons revealed co-expression of Kv4.2 and Kv4.3 subunits in most neurons (Fig. 2A). In comparison Kv4.1 subunits were expressed only at low levels in preoptic neurons (see below). Since Kv4.2 is a pERK substrate [19] we tested the hypothesis that it plays a role in modulating the firing activity and I_A_ of preoptic neurons downstream of H3R. Thus we compared the effect of H3 agonist in acutely dissociated w-t and Kv4.2−/− preoptic neurons. The spontaneous pacemaking activity and responses to H3 agonist of dissociated w-t preoptic neurons were very similar with those recorded in w-t slices (4). The average firing rate of a subpopulation of w-t neurons was inhibited by 48±7% (n = 6 out of 14 GABAergic neurons tested) during R-α-methylhistamine (1 µM) incubation (Fig. 2 B), value similar with that measured in slices (P>0.2 unpaired, t-test). Dissociated Kv4.2−/− GABAergic neurons displayed spontaneous pacemaker activity and action potential parameters similar with those of w-t cells (Table 1). The resting membrane potential (measured in TTX) was slightly more depolarized in Kv4.2−/− cells (Table 1). A striking difference was that Kv4.2−/− GABAergic neurons displayed only very small responses to the H3 agonist (Fig. 3 C,D). The average firing rate decreased by 1±5% (n = 6 of 23 neurons studied). R-α-methylhistamine (1 µM) increased the firing rate in two Kv4.2−/− GABAergic neurons and decreased it in the others (n = 4). In the remaining 17 neurons the agonist was without effect. The percent reduction of firing rates was significantly lower in Kv4.2−/− neurons than in w-t neurons (unpaired t-test, P<0.05). To address this discrepancy we investigated the expression of H3R in Kv4.2−/− neurons by immunocytochemistry and single cell RT/PCR. GAD1 and H3R immunoreactivities colocalized in ∼50% of neurons studied (n = 64 out of 131) (Fig. 2B). As previously found in w-t neurons [4] H3R transcripts were detected in a subpopulation (5 out of 13 GAD1 positive neurons, i.e. 38%) of GABAergic neurons. We have also quantified the concentration of H3R transcripts in w-t and Kv4.2−/− mRNA extracted from preoptic tissue (n = 6 mice each). No difference in H3R transcript concentration (normalized to GAPDH) was observed between w-t and Kv4.2−/− RNA samples (P>0.2, t-test) (data not shown). We therefore hypothesized that the lack of sensitivity to H3R agonist of Kv4.2−/− neurons is due to differences in I_A_ or in their sensitivity to modulation by H3R activation.

**Table 1:** Properties of preoptic GABAergic w-t and Kv4.2−/− neurons.

Parameter	w-t	*n*	Kv4.2−/−	*n*
r.m.p. (mV)	−52.3±4.4	14	−50.6±5.1*	15
Rinput (MΩ)	407±62	14	427±53	15
S. f. r. (Hz)	10.2±3.7	14	9.4±3.5	15
AP amplitude (mV)	77.2±4.4	14	78.3±5.7	15
AP threshold (mV)	−43±3	14	−44±4	15
AP halfwidth (mV)	0.8±0.1	14	0.9±0.1	15
AHP amplitude (mV)	−17.8±3.1	14	−19.2±3.2	15
AHP time to peak (ms)	5.5±3.4	14	4.9±3.9	15
AHP decay (mV/ms)	0.23±0.22	14	0.25±0.2	15
Peak I_A_ density at 0 mV (pA/pF)	58.6±19.5	14	41.8±12.9*	15
I_SS_ density at 0 mV (pA/pF)	45.4±19.5	14	82.3±27.5**	15

Values are presented as mean ± S.D., * and ** indicate P<0.05 and P<0.01, respectively (unpaired t-tests), (n)-number of cells.

A marked difference of voltage-gated K^+^ currents recorded in Kv4.2−/− GABAergic neurons was that the I_DR_ had larger amplitude than in w-t GABAergic neurons (Fig. 3A, D). We then tested the K^+^ current sensitivity to 4-AP (0.25 mM) and TEA (5 mM), blockers for Kv1 and Kv3 subunit families, respectively. We found that only the I_DR_ component was sensitive to either blocker in w-t and Kv4.2−/− GABAergic neurons. Combining the two blockers provided us with an effective means of separating I_A_ from the I_DR_ (Fig. 3A). Surprisingly, the I_A_ current density (peak amplitude/cell capacitance) was only 14% smaller in the transgenic (P<0.05, t-test, n = 14 and n = 15 w-t and Kv4.2−/− neurons, respectively). The voltage–dependence of activation (Fig. 3C, D) and inactivation (-Fig. 3E, F) were similar in w-t and Kv4.2−/− GABAergic neurons. The I_DR_ density was 81% larger (P<0.01 unpaired t-test, n = 14 and n = 15 w-t and Kv4.2−/− neurons, respectively) in Kv4.2−/− neurons (Fig. 3A, D).

We have also compared several electrical characteristics (membrane potential, firing rate, action potential characteristics, I_A_ current density, etc) of w-t GABAergic neurons in which I_A_ was potentiated by the H3 agonist (n = 6) with those of a group of w-t GABAergic neurons in which the current was not changed (n = 8). No differences were found between the two groups (P>0.1, t-tests) and therefore the data were pooled (Table 1).

### Activation of H3R specifically modulates I_A_ conducted by Kv4.2-containing channels

**Figure 4:**
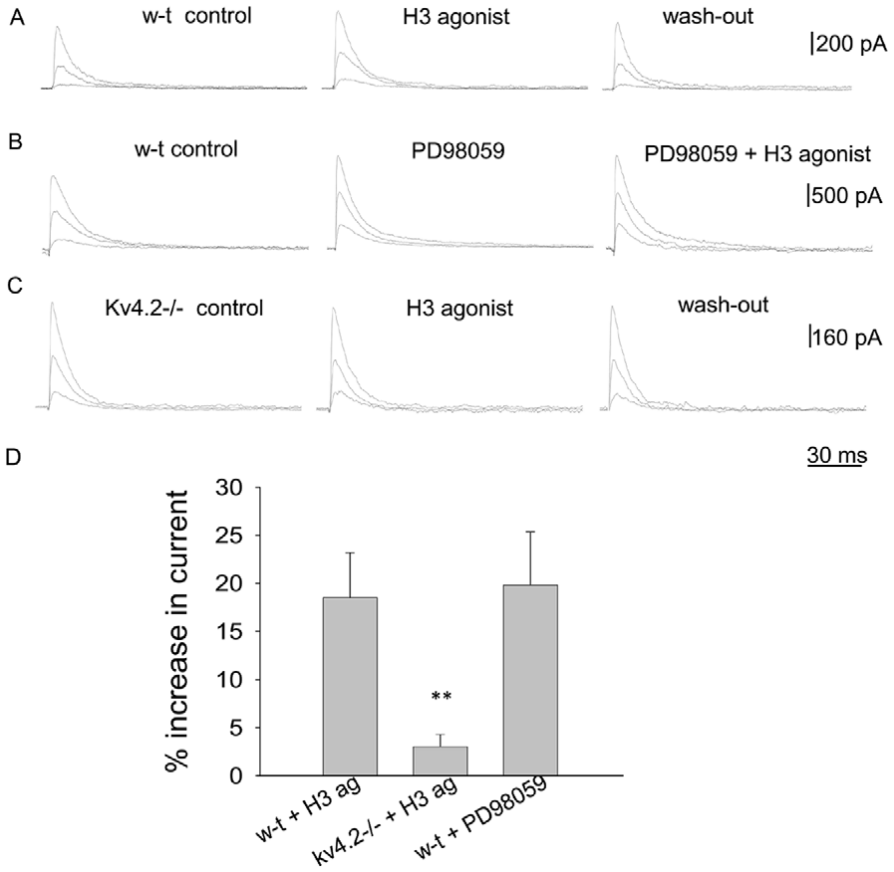
Effects H3 agonist on I_A_ in w-t and Kv4.2−/− GABAergic preoptic neurons. A,B. I_A_ recorded from a w-t neuron (A) and a Kv4.2−/− neuron (C) in control (left), after 5 min incubation with R-α-methylhistamine (1 µM) (middle) and after washout of the agonist (right). B. I_A_ recorded from a w-t neuron in control (left), after 5 min incubation with PD98059 (10 µM) (middle) and after subsequent 5 min incubation with R-α-methylhistamine (1 µM) (right). A–D. The currents were elicited by the voltage steps to −40, −30 and −20 mV from a holding potential of −110 mV. The extracellular solution contained TTX (1 µM), TEA (5 mM) and 4-AP (0.25 mM). E. Bar chart of the percentage increase in the peak I_A_ amplitude (at −20 mV) test potential by *R*-α-methylhistamine (1 µm). ** indicates statistical significance of P<0.01 when compared with the other 3 columns (ANOVA, Kruskal-Wallis with Dunn's post test). The bars represent averages ±S.D.

Since I_A_ properties were little changed in Kv4.2−/− neurons we then questioned whether the lack of effect of the H3 agonist on the spontaneous firing activity of preoptic GABAergic neurons was due to a lack of effect on the I_A_ amplitude, or to other compensatory mechanisms. A decrease in the level of pERK1/2 mimics the effects of H3R activation [4] on the firing activity of preoptic neurons therefore we also studied the effects of the MEK-1 inhibitor PD98059 (10 µM) on the I_A_ properties. As described above, R-α-methylhistamine (1 µM) increased I_A_ in 35% of w-t GABAergic neurons studied by 18±5% (n = 10 out of 29). In contrast, the agonist increased I_A_ by 2±5% (n = 8 out of 26) in Kv4.2−/− GABAergic neurons at −20 mV test potential (Fig. 3 A,C,D). R-α-methylhistamine (1 µM) decreased I_A_ in two Kv4.2−/− GABAergic neurons and increased the current in the others (n = 6). PD98059 (10 µM) mimicked the action of the H3 agonist in w-t neurons: it increased I_A_ by 20±6% (n = 5 out of 7) (Fig. 4 B, D). The H3 agonist did not have any effect on the I_A_ amplitude in neurons pretreated with PD98059 (Fig. 4 B, D). We also tested for possible effects of H3R activation on the I_DR_ in Kv4.2−/− neurons. As described above for w-t neurons, R-α-methylhistamine (1 µM) was without effect on I_DR_ (n = 12).

**Figure 5:**
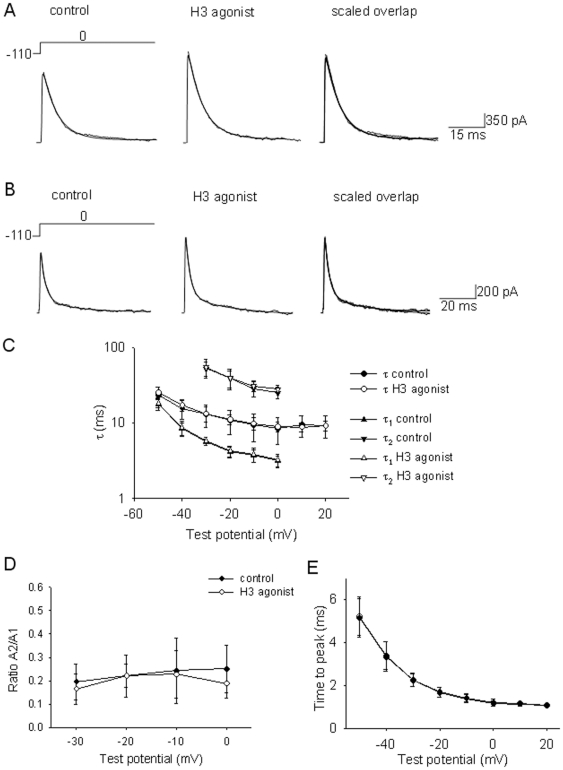
Lack of effect of H3 agonist on I_A_ kinetics in GABAergic preoptic neurons. A,B. I_A_ recorded from two w-t neuron in control (left), after 5 min incubation with R-α-methylhistamine (1 µM) (middle) and their scaled overlap (right). The currents were elicited by the voltage steps to 0 mV from a holding potential of −110 mV. The extracellular solution contained TTX (1 µM), TEA (5 mM) and 4-AP (0.25 mM). The traces in A were fitted by a single exponential decay A*e(−t/τ). T was 10.3 ms in the control and 10.6 during H3 agonist incubation. The currents in B were fitted with a biexponential decay A_1_*e(−t/τ_1_)+A_2_*e(−t/τ_2_). T1 was 2.9 ms in the control and 3.0 ms during H3 agonist incubation. T2 was 29.4 ms in the control and 28.9 ms during H3 agonist incubation. C. Voltage-dependence of the time constants of I_A_ decay. Some cells displayed monoexponential decay (τ) (n = 6) or biexponential decay (τ_1_ and τ_2_) (n = 4) in control (black) and during H3 agonist incubation (white). The data points at +10 and +20 mV are from 4 neurons dispaying monoexponential decay. D. Voltage-dependence of the A2/A1 ratio of neurons that displayed biexponential decays (n = 4) in control (•), and during H3 agonist incubation (○). E. Voltage-dependence of the time to peak of I_A_ (n = 10) in control (•), and during H3 agonist incubation (○). C–E. Data are presented as averages ± S.D.

We have also studied possible effects on I_A_ kinetics in neurons in which the current was enhanced by the H3 agonist. I_A_ decay was fitted by a single exponential decay in 6 out of 10 neurons studied (Fig. 5A). The remaining 4 neurons displayed a biexponential decay (Fig. 5B). The H3 agonist did not change the time constants of the exponential decays in any of the 10 neurons (Fig. 5A–C). The agonist also did not change the relative weights of the fast and slow components in bi-exponential decays (Fig. 5D). Finally, the H3 agonist did not change the time to peak in any neuron (n = 10, Fig. 5E).

### Molecular identity of the K^+^ channels conducting I_A_ in Kv4.2−/− GABAergic preoptic neurons

I_A_ was insensitive to 4-AP and TEA in w-t and Kv4.2−/− GABAergic preoptic neurons while I_DR_ was fully blocked by the combined blockers (Fig. 3A,B). We then studied the effect of PaTX1 (600 nM) on the I_A_ of Kv4.2−/− GABAergic neurons. The blocker reduced I_A_ by 70±13% (n = 5), value similar with that observed for w-t GABAergic neurons (P>0.5, unpaired t-tests). These results indicated the involvement of Kv4.1 and/or Kv4.3 subunits in the generation of I_A_ in Kv4.2−/− neurons.

**Figure 6:**
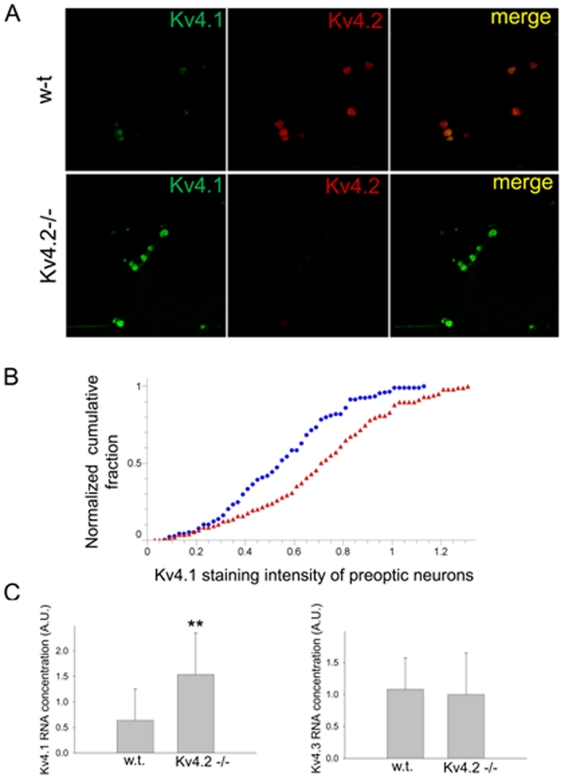
Upregulation of the level of expression of Kv4.1 subunits in Kv4.2−/− preoptic neurons. A. Single channel confocal images showing immunoreactivity to Kv4.1 (*Left*, green) and Kv4.2 (*Middle*, red) and their superimposition (*Right*, merge) obtained from dissociated w-t (upper row) and Kv4.2−/− (lower row) preoptic neurons. B. Cumulative histograms of the Kv4.1 staining intensity in w-t (blue circles, n = 708 neurons) and Kv4.2−/− neurons (red triangles, n = 811 neurons). The distributions were significantly different (P<0.01, Kolmogorov-Smirnov test). C. qPCR analysis of Kv4.1 (left) and Kv4.3 gene (right) expression in preoptic area from w-t and Kv4.2−/− mice (n = 6 mice each). For normalization GAPDH was selected as housekeeping gene. Bars represent averaged normalized concentrations ± SD. ** represents significant differences between w-t and Kv4.2−/− groups P<0.01 (t-test).

To clarify the expression of the Kv4 subunits we have performed immunocytochemistry experiments on w-t and Kv4.2−/− preoptic neurons. Kv4.2 and Kv4.3 were highly expressed in w-t neurons (Fig. 2A) while Kv4.1 was expressed at low levels (Fig. 6A). In contrast, the transgenic preoptic neurons the level of expression of Kv4.1 was high (Fig. 6A, B) while Kv4.3 expression levels remained the same. We have also carried out qPCR on RNA extracted from preoptic tissue from w-t and Kv4.2−/− mice (n = 6 mice each). After reverse transcription of RNA was performed, Kv4.1, Kv4.3 and GAPDH expression was measured using qPCR (Fig. 6C). When normalized with respect to GAPDH gene expression, levels of Kv4.1 were found to be ∼3 fold increased in the Kv4.2−/− samples when compared to the wild-type samples (t-test, P<0.01). In contrast, the levels for Kv4.3 transcripts appeared to decrease albeit the difference was not statistically significant (t-test, P>0.2) (Fig. 6C).

### Dynamic clamp simulation of an increase in I_A_ in w-t and Kv4.2−/− neurons

**Figure 7:**
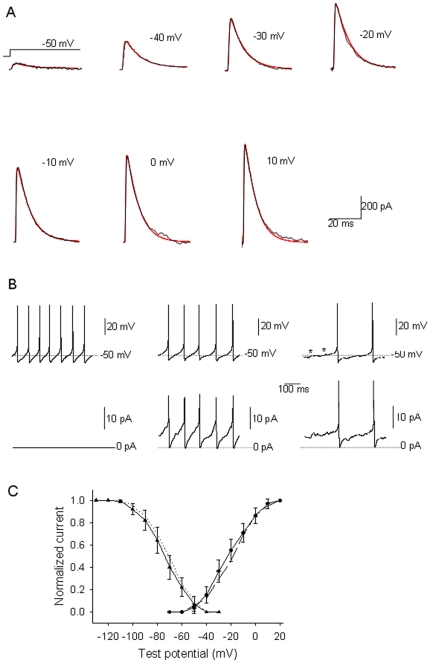
Dynamic clamp experiments reveal the role of I_A_ in the pacemaking activity of preoptic neurons. A. Comparison between the recorded I_A_ (black) and those generated by the corresponding model (red). The currents were elicited by step depolarizations from −110 mV holding potential to the indicated test potentials. B. Spontaneous firing activity in a preoptic neuron in control (left upper trace) and when the I_A_ current is increased by 10% (middle upper trace) or 20% (right upper trace). The lower traces represent the I_A_ injected by the dynamic clamp. Note the prolonged ramp-like potentials that precede a spike and the presence of subthreshold membrane potential oscillations (*) during dynamic clamp. C. Voltage-dependence of activation (•) and inactivation (▴) of I_A_ recorded from w-t neurons (data replotted from Fig. 3 C and F). The lines represent voltage-dependence of activation (dashed line) and inactivation (dotted line) of I_A_ generated by our model.

In order to determine whether an increase in I_A_ could account for the decrease in the spontaneous firing activity of dissociated preoptic neurons we carried out dynamic clamp experiments. We have first measured I_A_ in a neuron as described above (in the presence of TTX, 4-AP and TEA) and then washed out the drugs to recover the spontaneous firing activity. Meanwhile we have determined the Hodgkin-Huxley model that fitted the currents (see Methods) (Fig. 7A). Using dynamic clamp we have added an I_A_ component with a maximum conductance of 10% or 20% of gKa (according to the model calculated for the same cell). Increasing I_A_ by 10% and 20% resulted in a decrease of the average firing rate by 19% and 52%, respectively (Fig. 7B). The ramp-like potentials preceding the action potentials were prolonged during dynamic clamp. We have also noticed sub-threshold oscillations in the membrane potential during dynamic clamp (* in Fig. 7B). Results in 5 preoptic neurons yielded similar results, namely a decrease of 18±5% (n = 6) and 46±5% (n = 6) when I_A_ was augmented by 10 and 20%, respectively. Since the Kv4.2−/− preoptic neurons presented electrical remodeling, i.e. smaller I_A_ and much larger I_DR_ (see above), we then questioned whether their firing rates would be influenced by an increase in I_A_. Results in 5 Kv4.2−/− neurons studied were similar to those in w-t neurons: a decrease of 20±4% (n = 4, P>0.5, unpaired t-test) and 49±7% (n = 4, P>0.5, unpaired t-test) when I_A_ current was augmented by 10 and 20%, respectively.

### Effects of intra-MnPO injection H3R agonist on core body temperature and motor activity in w-t and Kv4.2−/− mice

**Figure 8:**
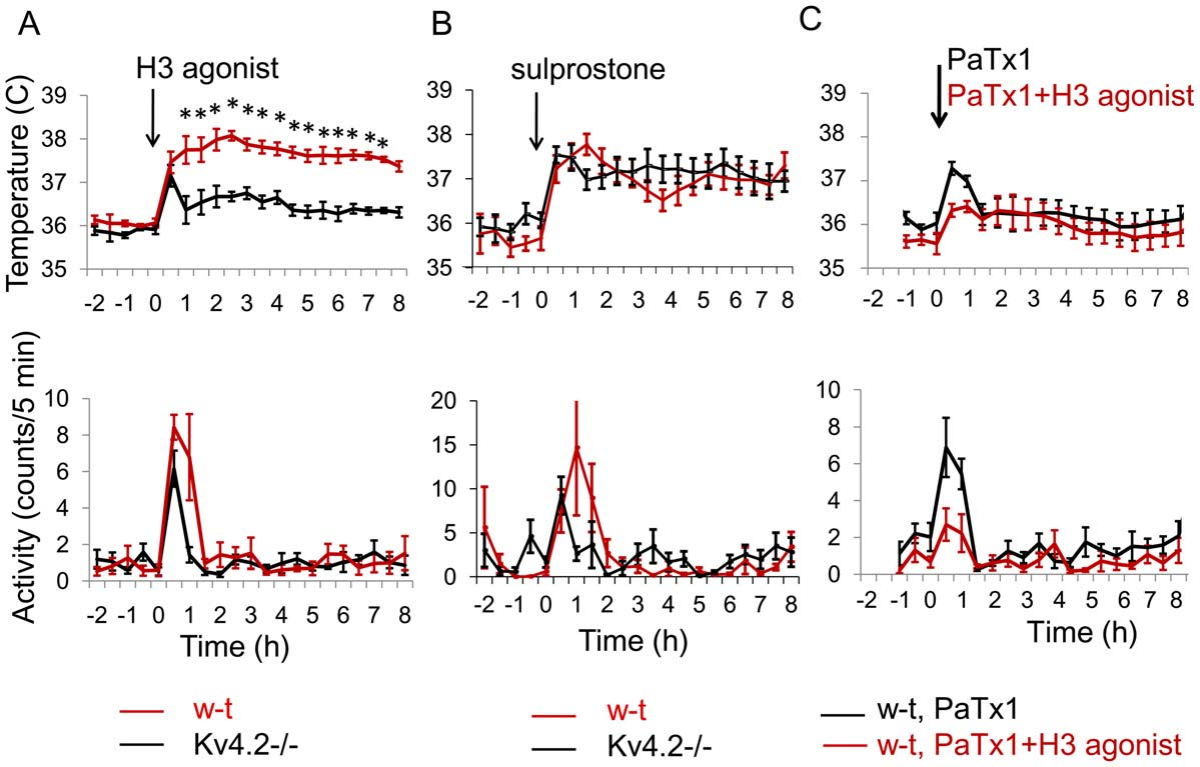
Responses to intra-MnPO injection of H3R agonist and sulprostone in w-t and Kv4.2−/− mice. A. Intra-MnPO injection of H3R agonist (10 µM, 0.2 µl) induced hyperthermia (upper panel) and did not affect motor activity (MA)(lower panel) in w-t and Kv4.2−/− mice (red and black traces, respectively). The CBT response was significantly smaller in Kv4.2−/− mice (two-way repeated measures ANOVA, followed by t-tests for each time point, * P<0.05). B. Intra-MnPO injection of EP1/3 agonist sulprostone (10 µM, 0.2 µL) induced hyperthermia (upper panel) and did not affect motor activity (MA)(lower panel) in w-t and Kv4.2−/− mice. The responses were not different in the w-t and Kv4.2−/− mice (P>0.4 two-way repeated measures ANOVA). C. Intra-MnPO injection of PaTx1(800 nM, 0.2 µL) or PaTx1+H3 agonist (800 nM and 10 µM, 0.2 µL) induced a slight hyperthermia of 0.4 and 0.6°C, respectively, (upper panel) and did not affect motor activity (MA)(lower panel) in w-t and Kv4.2−/− mice. The responses were not different (P>0.2 two-way repeated measures ANOVA). A–C. The line graphs represent averages±S.D. (n = 6 mice) through the 10 h recording period. Experiments were carried out in parallel in 2 groups of 6 mice for each treatment.

**Figure 9:**
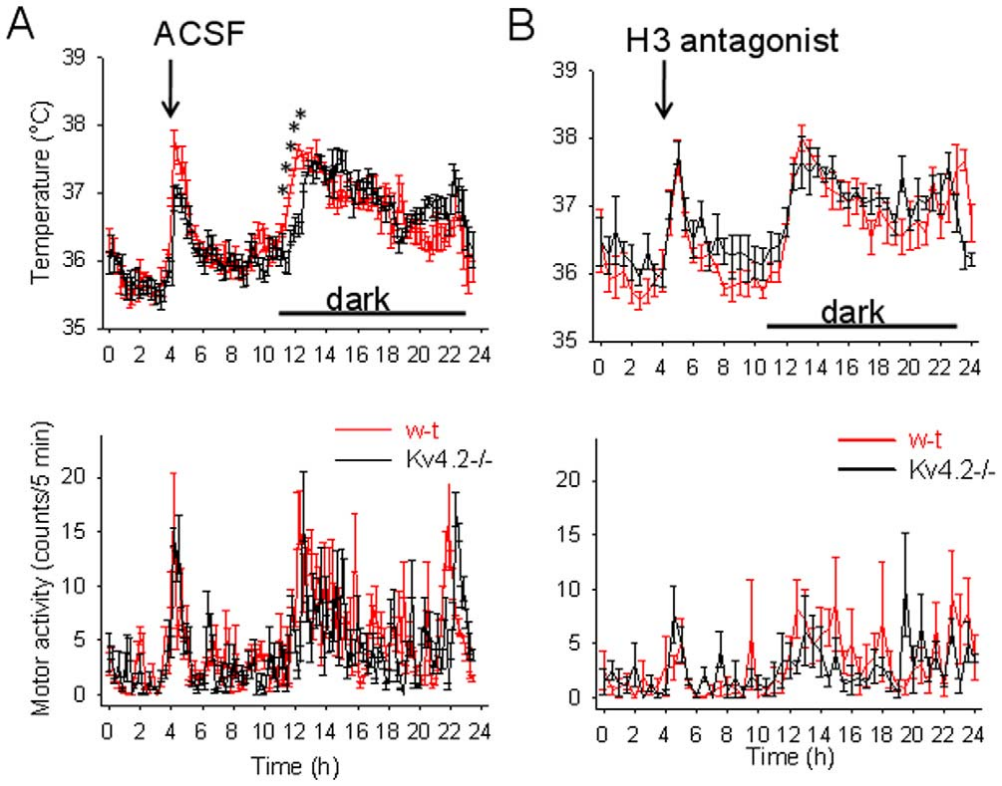
Circadian CBT and MA profiles and responses to H3 antagonist infusions. A. Intra-MnPO injection of aCSF (0.2 µL) had no effect on CBT (upper panel) or MA (lower panel) in w-t and Kv4.2−/− mice. The circadian CBT profile of Kv4.2−/− mice was shifted by 1 h (two-way repeated measures ANOVA followed by t-tests for each time point, * P<0.05). Data were pooled and averaged from 6 w-t (black) and 6 Kv4.2−/− (red) neurons. Data are plotted at 12 min intervals to emphasize the shift in circadian rhythm. The MA of Kv4.2−/− mice was not significantly different (two-way repeated measures ANOVA followed by t-tests for each time point, P>0.2). B. Intra-MnPO injection of H3 antagonist thioperamide (1 µM, 0.2 µl) delayed the CBT rise by 1 h in w-t (red) mice and had no effect in Kv4.2−/− mice (black). After thioperamide infusion the CBT profiles of w-t and Kv4.2−/− mice were not different (two-way repeated measures ANOVA followed by t-tests for each time point, P>0.1).

We have previously reported that histamine and the H3 agonist R-α-methylhistamine injected in the MnPO increase core body temperature (CBT) without changing motor activity (MA) [4]. Here we compared the effect of the H3 agonist (10 µM, 0.2 µL) in w-t and Kv4.2−/− mice. Experiments were carried out in parallel in 2 groups of 6 mice. The agonist induced a robust hyperthermia when injected in the MnPO (Fig. 8A, upper panel), an effect which was not accompanied by increased motor activity (Fig. 8A, lower panel), suggesting that it was due to changes in thermoregulation (e.g. brown adipose tissue thermogenesis). In contrast, the Kv4.2−/− mice developed a much smaller hyperthermia (two-way repeated measures ANOVA, P<0.01). Control aCSF injections (0.2 µL, control) did not result in hyperthermia in either strain of animals (Fig. 9A). As a positive control, we have also tested the ability of Kv4.2−/− mice to develop thermogenic responses by injecting a potent pyrogen, the EP1/3 prostanoid receptor agonist sulprostone (5 µM, 0.2 µL). The responses of Kv4.2−/− mice were indistinguishable from those of w-t mice (Figure 7B) and reached a maximum value of ∼2°C above baseline, proving that these transgenics are able of developing a full amplitude hyperthermia. We have tested then the effect of the H3 agonist when co-applied with PaTx1 (10 µM and 800 nM, respectively, in 0.2 µL final volume). In the presence of the toxin the effect of the agonist was abolished (Figure 8C), i.e. the responses to PaTx1 (800 nM, 0.2 µL) and PaTx1+ H3 agonist were not different (two-way repeated measures ANOVA, P>0.1).

**Figure 10:**
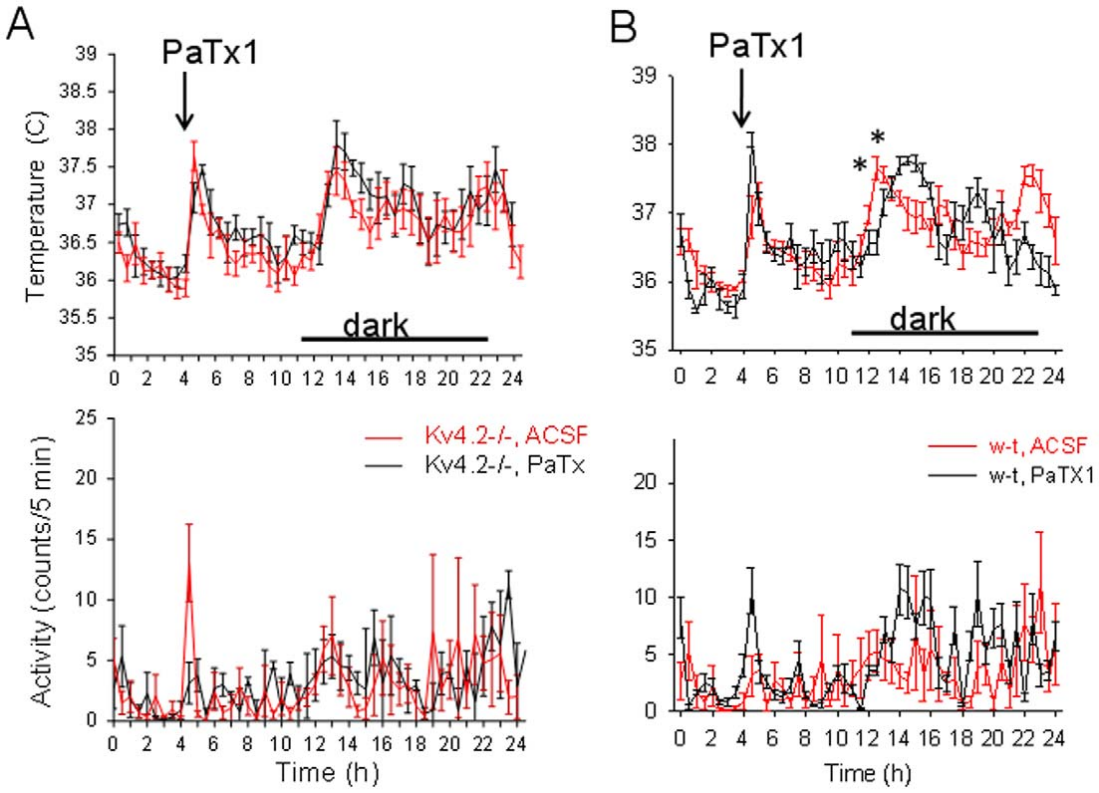
Intra-MnPO injection of PaTx1 shifts the CBT circadian profile in w-t mice. A. CBT (upper panel) and MA (lower panel) responses to intra-MnPO injection of PaTx1 (800 nM, 0.2 µL) (black) and aCSF (0.2 µL) (red) injections in Kv4.2−/− mice B. CBT (upper panel) and MA (lower panel) responses to intra-MnPO injection of PaTx1 (800 nM, 0.2 µL) (black) and aCSF (0.2 µL) (red) injections in w-t mice. The toxin induced a ∼1 h delay in the CBT rise associated with the active phase (two-way repeated measures ANOVA followed by t-tests for each time point, * P<0.05) and did not affect motor activity (two-way repeated measures ANOVA followed by t-tests for each time point, P>0.3). A–B. The line graphs represent averages±S.D. through the 24 h recording period. Experiments were carried out in parallel in 2 groups of 6 and Kv4.2−/− and w-t mice, respectively.

Histamine has been reported to be a potent modulator of circadian rhythms *in vivo* and *in vitro* (reviewed in [1]). We have first examined the circadian pattern in CBT and MA in w-t and Kv4.2−/− mice. The only difference observed was that in the transgenic the rise in CBT associated with the onset of the dark phase of the cycle was delayed by ∼1 h (* in Fig. 9A). Similar observations were made in 2 other sets of w-t and Kv4.2−/− mice (n = 6 each). Therefore, to determine whether the circadian rhythm in CBT is influenced by preoptic histamine signaling, we have injected the H3 antagonist thioperamide (1 µM, 0.2 µL, n = 6 each) intra-MnPO in w-t and Kv4.2−/− mice. The antagonist delayed the rise in CBT by ∼1 h in w-t mice but was without effect in the transgenic (Fig. 9B), i.e. after thioperamide infusion the CBT profiles of the w-t and Kv4.2−/− mice were overlapping. In contrast, the antagonist had no effect on the MA of the mice (two-way repeated measures ANOVA, P>0.4). We then studied the role of Kv4 subunits expressed by preoptic neurons in the circadian CBT profile by injecting intra-MnPO the Kv4 selective toxin PaTx1 (800 nM, 0.2 µL). The toxin had no effect on the circadian rhythm of CBT in Kv4.2−/− mice (Fig. 10A) but shifted the rising phase in CBT by ∼1 h in w-t mice, the profile becoming similar to that of Kv4.2−/− mice (Fig. 10B). The toxin did not affect the MA of either w-t or Kv4.2−/− mice (Fig. 10B) when injected intraMnPO.

## Discussion

### H3R activation decreases the firing rate of preoptic GABAergic neurons by increasing I_A_

We have previously reported that histamine inhibits the firing rate of preoptic GABAergic neurons by activating postsynaptic H3Rs and subsequent decrease in the level of pERK1/2 [4]. In this study we establish that activation of H3Rs decreases the spontaneous firing rate also in acutely dissociated neurons and that this effect is associated with a ∼20% increase in I_A_. These findings confirm the postsynaptic nature of histamine modulation of preoptic GABAergic neurons. The percentage value of the I_A_ potentiation is similar with that reported by others in studies where the current was found to be increased in response to neuromodulators [20] or in response to direct inhibition of kinases [21], i.e. in the range 10–30%. It is interesting to note that also in these preparations I_A_ is conducted by Kv4 subunits. This relatively modest enhancement of I_A_ resulted in robust reduction in firing rate (∼2.2% decrease in firing rate per 1% increase in current) suggesting that this mechanism is a powerful mechanism for controlling neuronal firing rates. This value is similar with that reported for arcuate neurons (∼2% decrease in firing rate per 1% increase in current) [20] and comparable with that reported in midbrain dopaminergic neurons (∼1.8% decrease in firing rate per 1% increase in current) [22].

Using dynamic clamp we also demonstrate that an enhancement (by 20%) of I_A_ is sufficient for decreasing the spontaneous firing rate of the neuron by a similar extent to that induced by H3R agonist. Previous studies have documented the role of I_A_ in spike frequency adaptation in response to depolarizing current injections [23], [24] and in regulating the rate of pacemaking activity [20], [25], [26]. Our data further confirm the role of somatic I_A_ in the regulation of “pacemaker” firing activity by determining the length of the depolarizing ramps preceding a spike [25], [27]. It has been shown, using siRNA knockdown, that Kv4 subunits are necessary for generating subthreshold membrane potential oscillations [28]. Here we prove that, conversely, increasing I_A_ by dynamic clamp results in such events occurring more frequently.

### Signaling pathway activated downstream of H3R

The H3 agonist-induced I_A_ enhancement was mimicked by decreasing the pERK1/2 level pharmacologically. Incubation with the MEK-1 inhibitor also occluded additional effects of the agonist confirming the central role played by ERK1/2 in this signaling mechanism. We have also established that the Kv4.2 subunit, a known ERK1/2 substrate [19], [29] is required for H3 agonist-induced augmentation of I_A_. This finding was surprising since also the Kv4.1 and Kv4.3 subunits present ERK consensus sequences on the intracellular C-terminus domain. These observations suggest a specific interaction of the Kv4.2 subunit with the H3/pERK1/2 pathway.

### Electrical remodeling of preoptic Kv4.2−/− neurons

The I_A_ and the action potential characteristics were not changed in Kv4.2−/− neurons, in spite of the fact that Kv4.2 subunits are highly expressed in w-t preoptic GABAergic neurons. We found that Kv4.2−/− neurons presented a threefold increase in the expression of the Kv4.1 subunit. This was unexpected since, in contrast to Kv4.2 and Kv4.3, the Kv4.1 subunit is expressed only at very low levels in preoptic neurons (and generally in the brain). Another surprising finding was that while I_A_ density was little changed (14% smaller) in Kv4.2−/− neurons, the I_DR_ density was 81% higher than that of w-t neurons. Interestingly, a similar upregulation of I_DR_ was noticed also in Kv4.2−/− cortical neurons [30], however, the role played by the upregulated I_DR_ in Kv4.2−/− neurons remains to be determined.

### Role of preoptic histamine signaling in thermoregulation

Preoptic GABAergic neurons that express H3Rs contribute to the inhibitory tone sent to neurons in the DMH and/or rRPA that control thermogenesis (reviewed in [3]). Thus, in the presence of H3 agonist a decrease in the frequency of their tonic firing results in diminished inhibition of downstream thermoregulatory neurons and increased thermogenesis. Although Kv4.2−/− mice were able to develop full size hyperthermia when challenged with EP1/3 agonist (intra-MnPO), they developed much smaller responses than the w-t mice in response to intra-MnPO infusion of H3 agonist. In the presence of the Kv4 specific toxin PaTx1 the H3 agonist was without effect. Together with results *in vitro* discussed above (with the caveat that those have been obtained in younger animals) our findings indicate a central role of the Kv4.2 subunit in the H3R signaling pathway in preoptic GABAergic neurons.

Since the hypothalamic concentration of histamine displays a circadian rhythm [31] and increases during arousal [32], [33] we questioned the role of H3R signaling in this rhythm. We found that intraMnPO infusion of thioperamide delayed the rise in CBT associated with the active phase by ∼1 h, strongly suggesting a role of histamine acting at H3Rs. Interestingly, Kv4.2−/− mice presented a similar shift in their circadian CBT profile in agreement with a possible disruption of H3R signaling. These results indicate for the first time a role of histamine signaling in MnPO neurons for the control of the circadian profile in CBT. However, we should note that thioperamide did not block the increase in CBT associated with the active phase, indicating that multiple mechanisms, possibly sequential, exist for the control of this rhythm. Neither the H3 agonist nor the H3 antagonist (administerd intra-MnPO) changed the motor activity of w-t or Kv4.2−/− mice, confirming that histamine does not influence the MA circadian rhythm as previously reported [34]. While CBT and MA circadian rhythms are tightly coupled in normal conditions, they are regulated by distinct anatomical substrates [35], [36] and/or signaling mechanisms [37]. Our results indicate that histamine acting at H3Rs in preoptic GABAergic neurons provides a mechanism of selective regulation of the circadian rhythm of CBT at the beginning of the active phase.
